# 4-(4-Chloro­phen­yl)-4-hy­droxy­piperidinium maleate maleic acid solvate

**DOI:** 10.1107/S1600536810026917

**Published:** 2010-07-14

**Authors:** Jerry P. Jasinski, Albert E. Pek, B. P. Siddaraju, H. S. Yathirajan, B. Narayana

**Affiliations:** a Department of Chemistry, Keene State College, 229 Main Street, Keene, NH 03435-2001, USA; b Department of Studies in Chemistry, V.V Puram College of Science, Bangalore 560 004, India; c Department of Studies in Chemistry, University of Mysore, Manasagangotri, Mysore 570 006, India; d Department of Studies in Chemistry, Mangalore University, Mangalagangotri, 574 199, India

## Abstract

In the cation of the title compound, C_11_H_15_ClNO^+^·C_4_H_3_O_4_^−^·C_4_H_4_O_4_, the dihedral angle between the mean planes of the chlorine-substituted aromatic ring and the 4-hy­droxy­piperidinium ring (C–C–C–C–C–N) is 61.9 (8)°. Intra­molecular O—H⋯O and inter­molecular O—H⋯O and N—H⋯O hydrogen bonding, as well as weak π-stacking inter­actions [centroid–centroid distance = 3.646 (5) Å] help to establish the packing.

## Related literature

For the synthesis and biological activity of uncondensed cyclic derivatives of piperidine, see: Vartanyan (1984 ▶). For related structures, see: James & Williams (1974 ▶); Bertolasi *et al.* (1980 ▶); Dawson *et al.* (1986 ▶); Vyas *et al.* (1999 ▶); Kiang *et al.* (2003 ▶); Trask *et al.* (2005 ▶); Mohamed *et al.* (2009 ▶); Dutkiewicz *et al.* (2010 ▶); Fun *et al.* (2010 ▶); Jasinski *et al.* (2010 ▶). For bond-length data, see: Allen *et al.* (1987 ▶). For puckering parameters, see: Cremer & Pople (1975 ▶).
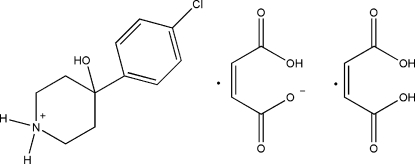


## Experimental

### 

#### Crystal data

C_11_H_15_ClNO^+^·C_4_H_3_O_4_^−^·C_4_H_4_O_4_
*M* *_r_* = 443.83
Monoclinic, 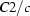

*a* = 19.282 (7) Å
*b* = 7.867 (3) Å
*c* = 25.115 (9) Å
β = 91.545 (5)°
*V* = 3808 (2) Å^3^
*Z* = 8
Mo *K*α radiation
μ = 0.26 mm^−1^
*T* = 100 K
0.52 × 0.41 × 0.39 mm

#### Data collection

Bruker APEXII CCD diffractometer
Absorption correction: multi-scan (*SADABS*; Bruker, 2008 ▶) *T*_min_ = 0.878, *T*_max_ = 0.907
18187 measured reflections
5841 independent reflections
5194 reflections with *I* > 2σ(*I*)
*R*_int_ = 0.021

#### Refinement

*R*[*F* ^2^ > 2σ(*F* ^2^)] = 0.034
*wR*(*F* ^2^) = 0.092
*S* = 1.03
5841 reflections
292 parameters
H atoms treated by a mixture of independent and constrained refinement
Δρ_max_ = 0.48 e Å^−3^
Δρ_min_ = −0.23 e Å^−3^

### 

Data collection: *APEX2* (Bruker, 2008 ▶); cell refinement: *SAINT* (Bruker, 2008 ▶); data reduction: *SAINT*; program(s) used to solve structure: *SHELXS97* (Sheldrick, 2008 ▶); program(s) used to refine structure: *SHELXL97* (Sheldrick, 2008 ▶); molecular graphics: *SHELXTL* (Sheldrick, 2008 ▶); software used to prepare material for publication: *SHELXTL*.

## Supplementary Material

Crystal structure: contains datablocks global, I. DOI: 10.1107/S1600536810026917/wm2372sup1.cif

Structure factors: contains datablocks I. DOI: 10.1107/S1600536810026917/wm2372Isup2.hkl

Additional supplementary materials:  crystallographic information; 3D view; checkCIF report

## Figures and Tables

**Table 1:** Hydrogen-bond geometry (Å, °)

*D*—H⋯*A*	*D*—H	H⋯*A*	*D*⋯*A*	*D*—H⋯*A*
O1*C*—H1*C*⋯O2*B*^i^	0.82	1.97	2.7852 (13)	171
O1*A*—H2*A*⋯O1*B*^ii^	0.90 (2)	1.68 (2)	2.5546 (13)	162 (2)
N1*C*—H13*C*⋯O3*B*^iii^	0.890 (17)	1.954 (18)	2.8328 (14)	168.6 (16)
N1*C*—H14*C*⋯O2*A*^iv^	0.887 (17)	2.087 (17)	2.9144 (15)	154.9 (15)
O4*A*—H1*A*⋯O3*A*	0.91 (2)	1.65 (2)	2.5531 (13)	173.7 (19)
O3*B*—H1*B*⋯O4*B*	1.18 (2)	1.23 (2)	2.4108 (12)	177 (2)

Symmetry codes: (i) 
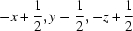
; (ii) 
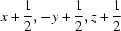
; (iii) 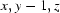; (iv) 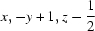.

**Fig. 1.**
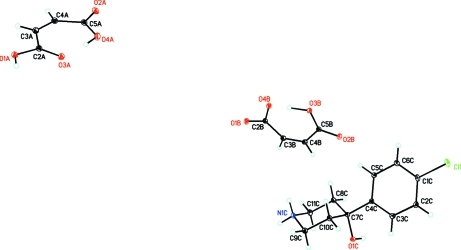
Molecular structures of the C11H15ClNO+, C4H3O4- and C4H4O4 entities, showing the atom labeling scheme and 30% probability displacement ellipsoids.

**Fig. 2.**
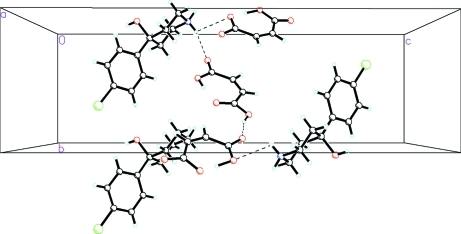
Packing diagram of the title compound C11H15ClNO+, C4H3O4-, C4H4O4, viewed down [100].

## supplementary crystallographic information

### Comment

4-(4-Chlorophenyl)-4-hydroxypiperidine is used as an intermediate for the synthesis of pharmaceuticals such as haloperidol (a neuroleptic drug used to treat patients with psychotic illnesses, extreme agitation, or Tourette's syndrome) and loperamide which is a synthetic piperidine derivative and a drug effective against diarrhea resulting from gastroenteritis or inflammatory bowel disease. A review on the synthesis and biological activity of uncondensed cyclic derivatives of piperidine is reported (Vartanyan, 1984). A study of the structural chemistry of maleic acid and related substances arises from the fact that these systems possess short but highly strained hydrogen bonds (James & Williams, 1974). The crystal structures of maleic acid (James & Williams, 1974), carbinoxamine maleate (Bertolasi *et al.*, 1980), [2-(2,2-dicyclohexylethyl) piperidine] maleate (Dawson *et al.*, 1986), domeperidone maleate (Vyas *et al.*, 1999), enalapril maleate (Kiang *et al.*, 2003), 1:1 co-crystal of caffeine with maleic acid (Trask *et al.*, 2005), 4-dimethylaminopyridinium maleate (Mohamed *et al.*, 2009), 4-(4-chlorophenyl)piperidin-4-ol (Dutkiewicz *et al.*, 2010), bis[4-(4-chlorophenyl)-4-hydroxypiperidinium] dipicrate dimethyl sulfoxide solvate (Fun *et al.*, 2010) and trimipraminium maleate (Jasinski *et al.*, 2010) have been reported. In view of the importance of salts of piperidines, this paper reports the crystal structure of the title compound, C_11_H_15_Cl N O^+^, C_4_H_3_O_4_^-^, C_4_H_4_O_4_.

The asymmetric unit of the title compound (Fig.1) contains one 4-(4-chlorophenyl)-4-hydroxypiperidinium cation, one maleate anion, and one maleic acid molecule. The protonated 4-hydroxypiperidinium cation is in a chair conformation (puckering parameters Q, θ, and φ = 0.576 (2) Å, 179.0 (8)° and 159.955 (0)°, respectively; (Cremer & Pople, 1975). For an ideal chair θ has a value of 0 or 180°). Bond distances and angles are in normal ranges (Allen *et al.*, 1987). The dihedral angle between the mean planes of the piperidinium ring in the cation (C7C/C8C/C9C/C10C/C11C/N1C) and the benzene ring (C1—C6) is 61.9 (8)°. Strong intramolecular O—H···O and intermolecular O—H···O, N—H···O hydrogen bonding interactions (Table 1, Fig. 2) dominate the crystal packing which leads to the formation of chains along [010]. In addition, weak π-stacking intermolecular interactions occur between symmetry related benzene rings (Table 2) which also influence the crystal packing.

### Experimental

4-(4-chlorophenyl)-piperidin-4-ol (2.2 g, 0.01 mol) and maleic acid (1.16 g, 0.01 mol) were dissolved in 20 ml of methanol. The mixture was stirred for 30 minutes at 333 K. Then the solution was kept aside for 3 days at room temperature. Yellow crystals were obtained (m.p: 381–383 K) by slow evaporation of methanol solution.

### Refinement

The hydroxyl H atoms, H1A, H2A, H1B, H1C and N H atoms, H13C, H14C, were located by a Fourier map. These H atoms and the rest of the H atoms were then positioned geometrically and allowed to ride on their parent atoms with *X*—H lengths of 0.91Å (O4A), 0.90Å (O1A), 1.18Å (O3B), 0.82Å (O1C), 0.89–0.89Å (N1C), 0.93Å (CH) or 0.97Å (CH_2_). Isotropic displacement parameters for these atoms were set to 1.4–3.5 times (OH), 1.8 times (NH), 1.20 (CH) or 1.2 (CH_2_) times *U*_eq_ of the parent atom.

### Crystal data

**Table:**

C_11_H_15_ClNO^+^·C_4_H_3_O_4_^−^·C_4_H_4_O_4_	*F*(000) = 1856
*M**_r_* = 443.83	*D*_x_ = 1.548 Mg m^−^^3^
Monoclinic, *C*2/*c*	Mo *K*α radiation, λ = 0.71073 Å
Hall symbol: -C 2yc	Cell parameters from 8484 reflections
*a* = 19.282 (7) Å	θ = 2.6–31.3°
*b* = 7.867 (3) Å	µ = 0.26 mm^−^^1^
*c* = 25.115 (9) Å	*T* = 100 K
β = 91.545 (5)°	Block, yellow
*V* = 3808 (2) Å^3^	0.52 × 0.41 × 0.39 mm
*Z* = 8	

### Data collection

**Table:**

Bruker APEXII CCD diffractometer	5841 independent reflections
Radiation source: fine-focus sealed tube	5194 reflections with *I* > 2σ(*I*)
graphite	*R*_int_ = 0.021
ω scans	θ_max_ = 31.3°, θ_min_ = 1.6°
Absorption correction: multi-scan (*SADABS*; Bruker, 2008)	*h* = −27→27
*T*_min_ = 0.878, *T*_max_ = 0.907	*k* = −11→11
18187 measured reflections	*l* = −35→35

### Refinement

**Table:**

Refinement on *F*^2^	Primary atom site location: structure-invariant direct methods
Least-squares matrix: full	Secondary atom site location: difference Fourier map
*R*[*F*^2^ > 2σ(*F*^2^)] = 0.034	Hydrogen site location: inferred from neighbouring sites
*wR*(*F*^2^) = 0.092	H atoms treated by a mixture of independent and constrained refinement
*S* = 1.03	*w* = 1/[σ^2^(*F*_o_^2^) + (0.0463*P*)^2^ + 3.2429*P*] where *P* = (*F*_o_^2^ + 2*F*_c_^2^)/3
5841 reflections	(Δ/σ)_max_ = 0.001
292 parameters	Δρ_max_ = 0.48 e Å^−^^3^
0 restraints	Δρ_min_ = −0.23 e Å^−^^3^

### Special details

**Table:**

Geometry. All e.s.d.'s (except the e.s.d. in the dihedral angle between two l.s. planes) are estimated using the full covariance matrix. The cell e.s.d.'s are taken into account individually in the estimation of e.s.d.'s in distances, angles and torsion angles; correlations between e.s.d.'s in cell parameters are only used when they are defined by crystal symmetry. An approximate (isotropic) treatment of cell e.s.d.'s is used for estimating e.s.d.'s involving l.s. planes.
Refinement. Refinement of *F*^2^ against ALL reflections. The weighted *R*-factor *wR* and goodness of fit *S* are based on *F*^2^, conventional *R*-factors *R* are based on *F*, with *F* set to zero for negative *F*^2^. The threshold expression of *F*^2^ > σ(*F*^2^) is used only for calculating *R*-factors(gt) *etc*. and is not relevant to the choice of reflections for refinement. *R*-factors based on *F*^2^ are statistically about twice as large as those based on *F*, and *R*- factors based on ALL data will be even larger.

### Fractional atomic coordinates and isotropic or equivalent isotropic displacement parameters (Å^2^)

**Table:**

	*x*	*y*	*z*	*U*_iso_*/*U*_eq_	
Cl1	0.480203 (14)	0.66435 (4)	0.161617 (11)	0.02088 (7)	
O4B	0.16691 (4)	0.58804 (10)	0.50337 (3)	0.01619 (15)	
O3B	0.22900 (4)	0.66101 (10)	0.42453 (3)	0.01604 (15)	
C5B	0.20779 (5)	0.56565 (13)	0.38519 (4)	0.01359 (18)	
C4B	0.14848 (6)	0.44620 (14)	0.39300 (4)	0.01543 (19)	
H4B	0.1353	0.3844	0.3628	0.019*	
C2B	0.11909 (5)	0.47937 (13)	0.49149 (4)	0.01414 (19)	
C3B	0.11113 (6)	0.41304 (14)	0.43602 (4)	0.01520 (19)	
H3B	0.0747	0.3371	0.4304	0.018*	
O3A	0.46151 (4)	0.35880 (11)	0.96021 (3)	0.01793 (16)	
C2A	0.44308 (6)	0.35385 (13)	1.00654 (4)	0.01452 (19)	
C3A	0.38419 (6)	0.45056 (14)	1.02827 (4)	0.0163 (2)	
H3A	0.3766	0.4349	1.0644	0.020*	
C5A	0.33654 (6)	0.61984 (14)	0.94690 (4)	0.01610 (19)	
C4A	0.34039 (6)	0.55740 (14)	1.00297 (4)	0.0162 (2)	
H4A	0.3060	0.6007	1.0244	0.019*	
O1A	0.47414 (4)	0.26101 (10)	1.04317 (3)	0.01712 (16)	
O2A	0.29197 (4)	0.72438 (11)	0.93499 (3)	0.02023 (17)	
O1B	0.07936 (4)	0.42630 (10)	0.52580 (3)	0.01788 (16)	
O2B	0.23403 (4)	0.57247 (10)	0.34108 (3)	0.01693 (16)	
C4C	0.37887 (5)	0.24201 (13)	0.25343 (4)	0.01180 (18)	
C1C	0.44091 (5)	0.50191 (14)	0.19706 (4)	0.01430 (19)	
C6C	0.39980 (5)	0.54258 (13)	0.23944 (4)	0.01428 (19)	
H6C	0.3928	0.6554	0.2490	0.017*	
C5C	0.36902 (5)	0.41155 (13)	0.26767 (4)	0.01336 (18)	
H5C	0.3415	0.4374	0.2964	0.016*	
C3C	0.42116 (5)	0.20613 (14)	0.21058 (4)	0.01490 (19)	
H3C	0.4287	0.0936	0.2010	0.018*	
C2C	0.45221 (6)	0.33510 (14)	0.18203 (4)	0.0161 (2)	
H2C	0.4800	0.3100	0.1534	0.019*	
O1C	0.33643 (4)	−0.04881 (10)	0.24944 (3)	0.01551 (15)	
H1C	0.3122	−0.0211	0.2235	0.023*	
C10C	0.39685 (5)	0.03445 (13)	0.32796 (4)	0.01363 (18)	
H10A	0.4395	−0.0037	0.3123	0.016*	
H10B	0.4080	0.1291	0.3514	0.016*	
C8C	0.27733 (5)	0.14407 (13)	0.30800 (4)	0.01358 (18)	
H8C1	0.2841	0.2427	0.3307	0.016*	
H8C2	0.2444	0.1744	0.2797	0.016*	
C7C	0.34665 (5)	0.09495 (13)	0.28369 (4)	0.01154 (17)	
C9C	0.36671 (5)	−0.10930 (14)	0.36035 (4)	0.01464 (19)	
H9C1	0.3594	−0.2083	0.3379	0.018*	
H9C2	0.3989	−0.1401	0.3891	0.018*	
C11C	0.24805 (5)	−0.00126 (14)	0.34043 (4)	0.01492 (19)	
H11A	0.2054	0.0348	0.3566	0.018*	
H11B	0.2374	−0.0968	0.3172	0.018*	
N1C	0.29935 (5)	−0.05450 (12)	0.38279 (3)	0.01394 (17)	
O4A	0.38012 (5)	0.56565 (12)	0.91126 (3)	0.02079 (17)	
H13C	0.2810 (9)	−0.142 (2)	0.3999 (7)	0.026 (4)*	
H14C	0.3072 (8)	0.031 (2)	0.4050 (7)	0.024 (4)*	
H1A	0.4103 (10)	0.490 (3)	0.9264 (8)	0.042 (5)*	
H2A	0.5113 (11)	0.206 (3)	1.0307 (8)	0.049 (6)*	
H1B	0.1992 (12)	0.629 (3)	0.4638 (9)	0.059 (6)*	

### Atomic displacement parameters (Å^2^)

**Table:**

	*U*^11^	*U*^22^	*U*^33^	*U*^12^	*U*^13^	*U*^23^
Cl1	0.01862 (13)	0.02183 (14)	0.02233 (13)	−0.00193 (9)	0.00295 (9)	0.01083 (10)
O4B	0.0190 (4)	0.0170 (4)	0.0125 (3)	−0.0050 (3)	−0.0001 (3)	−0.0003 (3)
O3B	0.0197 (4)	0.0149 (4)	0.0136 (3)	−0.0050 (3)	0.0016 (3)	−0.0013 (3)
C5B	0.0153 (4)	0.0120 (4)	0.0134 (4)	0.0008 (3)	−0.0006 (3)	0.0011 (3)
C4B	0.0185 (5)	0.0139 (5)	0.0138 (4)	−0.0022 (4)	−0.0015 (4)	−0.0018 (3)
C2B	0.0158 (5)	0.0126 (4)	0.0139 (4)	−0.0002 (4)	−0.0009 (3)	0.0011 (3)
C3B	0.0172 (5)	0.0140 (5)	0.0143 (4)	−0.0022 (4)	−0.0017 (4)	−0.0009 (3)
O3A	0.0202 (4)	0.0188 (4)	0.0150 (3)	0.0024 (3)	0.0031 (3)	0.0004 (3)
C2A	0.0154 (5)	0.0122 (4)	0.0160 (4)	−0.0015 (3)	0.0007 (3)	−0.0003 (3)
C3A	0.0191 (5)	0.0153 (5)	0.0146 (4)	0.0008 (4)	0.0027 (4)	0.0000 (4)
C5A	0.0168 (5)	0.0136 (5)	0.0178 (5)	−0.0020 (4)	−0.0011 (4)	0.0008 (4)
C4A	0.0171 (5)	0.0145 (5)	0.0170 (5)	0.0005 (4)	0.0022 (4)	−0.0005 (4)
O1A	0.0181 (4)	0.0173 (4)	0.0159 (3)	0.0041 (3)	0.0010 (3)	0.0013 (3)
O2A	0.0195 (4)	0.0164 (4)	0.0246 (4)	0.0012 (3)	−0.0029 (3)	0.0038 (3)
O1B	0.0198 (4)	0.0188 (4)	0.0151 (3)	−0.0042 (3)	0.0025 (3)	0.0011 (3)
O2B	0.0191 (4)	0.0187 (4)	0.0131 (3)	−0.0003 (3)	0.0019 (3)	0.0003 (3)
C4C	0.0123 (4)	0.0121 (4)	0.0110 (4)	−0.0001 (3)	−0.0002 (3)	0.0005 (3)
C1C	0.0122 (4)	0.0171 (5)	0.0136 (4)	−0.0018 (4)	−0.0003 (3)	0.0057 (4)
C6C	0.0151 (4)	0.0124 (4)	0.0154 (4)	−0.0006 (4)	0.0004 (3)	0.0013 (3)
C5C	0.0152 (4)	0.0129 (4)	0.0122 (4)	−0.0001 (3)	0.0023 (3)	−0.0003 (3)
C3C	0.0155 (5)	0.0144 (5)	0.0149 (4)	0.0006 (4)	0.0018 (3)	−0.0003 (4)
C2C	0.0158 (5)	0.0197 (5)	0.0128 (4)	0.0010 (4)	0.0035 (3)	0.0013 (4)
O1C	0.0234 (4)	0.0113 (3)	0.0117 (3)	−0.0010 (3)	−0.0014 (3)	−0.0015 (3)
C10C	0.0133 (4)	0.0147 (5)	0.0129 (4)	0.0004 (3)	0.0004 (3)	0.0017 (3)
C8C	0.0133 (4)	0.0128 (4)	0.0147 (4)	0.0006 (3)	0.0014 (3)	0.0030 (3)
C7C	0.0139 (4)	0.0100 (4)	0.0107 (4)	−0.0004 (3)	0.0006 (3)	−0.0003 (3)
C9C	0.0161 (5)	0.0143 (5)	0.0135 (4)	0.0015 (4)	0.0005 (3)	0.0019 (3)
C11C	0.0133 (4)	0.0160 (5)	0.0154 (4)	−0.0015 (4)	0.0002 (3)	0.0033 (4)
N1C	0.0172 (4)	0.0136 (4)	0.0110 (4)	−0.0017 (3)	0.0011 (3)	0.0011 (3)
O4A	0.0230 (4)	0.0236 (4)	0.0157 (4)	0.0043 (3)	0.0010 (3)	0.0026 (3)

### Geometric parameters (Å, °)

**Table:**

Cl1—C1C	1.7424 (11)	C1C—C2C	1.3844 (16)
O4B—C2B	1.2865 (13)	C6C—C5C	1.3929 (14)
O4B—H1B	1.23 (2)	C6C—H6C	0.9300
O3B—C5B	1.2978 (13)	C5C—H5C	0.9300
O3B—H1B	1.18 (2)	C3C—C2C	1.3876 (15)
C5B—O2B	1.2315 (13)	C3C—H3C	0.9300
C5B—C4B	1.4972 (15)	C2C—H2C	0.9300
C4B—C3B	1.3403 (15)	O1C—C7C	1.4314 (12)
C4B—H4B	0.9300	O1C—H1C	0.8200
C2B—O1B	1.2408 (13)	C10C—C9C	1.5178 (15)
C2B—C3B	1.4917 (15)	C10C—C7C	1.5301 (15)
C3B—H3B	0.9300	C10C—H10A	0.9700
O3A—C2A	1.2265 (13)	C10C—H10B	0.9700
C2A—O1A	1.3072 (13)	C8C—C11C	1.5212 (15)
C2A—C3A	1.4830 (15)	C8C—C7C	1.5339 (15)
C3A—C4A	1.3396 (16)	C8C—H8C1	0.9700
C3A—H3A	0.9300	C8C—H8C2	0.9700
C5A—O2A	1.2209 (14)	C9C—N1C	1.4932 (14)
C5A—O4A	1.3153 (14)	C9C—H9C1	0.9700
C5A—C4A	1.4912 (16)	C9C—H9C2	0.9700
C4A—H4A	0.9300	C11C—N1C	1.4930 (14)
O1A—H2A	0.90 (2)	C11C—H11A	0.9700
C4C—C5C	1.3952 (15)	C11C—H11B	0.9700
C4C—C3C	1.3965 (14)	N1C—H13C	0.890 (17)
C4C—C7C	1.5255 (14)	N1C—H14C	0.887 (17)
C1C—C6C	1.3816 (15)	O4A—H1A	0.91 (2)
			
C2B—O4B—H1B	111.1 (10)	C1C—C2C—C3C	118.61 (10)
C5B—O3B—H1B	111.1 (11)	C1C—C2C—H2C	120.7
O2B—C5B—O3B	122.07 (10)	C3C—C2C—H2C	120.7
O2B—C5B—C4B	118.62 (9)	C7C—O1C—H1C	109.5
O3B—C5B—C4B	119.30 (9)	C9C—C10C—C7C	112.16 (9)
C3B—C4B—C5B	131.05 (10)	C9C—C10C—H10A	109.2
C3B—C4B—H4B	114.5	C7C—C10C—H10A	109.2
C5B—C4B—H4B	114.5	C9C—C10C—H10B	109.2
O1B—C2B—O4B	120.98 (10)	C7C—C10C—H10B	109.2
O1B—C2B—C3B	118.74 (10)	H10A—C10C—H10B	107.9
O4B—C2B—C3B	120.28 (9)	C11C—C8C—C7C	111.33 (9)
C4B—C3B—C2B	129.85 (10)	C11C—C8C—H8C1	109.4
C4B—C3B—H3B	115.1	C7C—C8C—H8C1	109.4
C2B—C3B—H3B	115.1	C11C—C8C—H8C2	109.4
O3A—C2A—O1A	123.13 (10)	C7C—C8C—H8C2	109.4
O3A—C2A—C3A	125.25 (10)	H8C1—C8C—H8C2	108.0
O1A—C2A—C3A	111.63 (9)	O1C—C7C—C4C	110.55 (8)
C4A—C3A—C2A	128.80 (10)	O1C—C7C—C10C	105.37 (8)
C4A—C3A—H3A	115.6	C4C—C7C—C10C	109.86 (8)
C2A—C3A—H3A	115.6	O1C—C7C—C8C	109.32 (8)
O2A—C5A—O4A	120.67 (10)	C4C—C7C—C8C	112.22 (8)
O2A—C5A—C4A	118.03 (10)	C10C—C7C—C8C	109.28 (8)
O4A—C5A—C4A	121.29 (10)	N1C—C9C—C10C	109.67 (9)
C3A—C4A—C5A	132.01 (10)	N1C—C9C—H9C1	109.7
C3A—C4A—H4A	114.0	C10C—C9C—H9C1	109.7
C5A—C4A—H4A	114.0	N1C—C9C—H9C2	109.7
C2A—O1A—H2A	112.2 (13)	C10C—C9C—H9C2	109.7
C5C—C4C—C3C	118.49 (9)	H9C1—C9C—H9C2	108.2
C5C—C4C—C7C	122.50 (9)	N1C—C11C—C8C	110.12 (9)
C3C—C4C—C7C	118.99 (9)	N1C—C11C—H11A	109.6
C6C—C1C—C2C	121.83 (9)	C8C—C11C—H11A	109.6
C6C—C1C—Cl1	119.35 (9)	N1C—C11C—H11B	109.6
C2C—C1C—Cl1	118.82 (8)	C8C—C11C—H11B	109.6
C1C—C6C—C5C	118.81 (10)	H11A—C11C—H11B	108.1
C1C—C6C—H6C	120.6	C11C—N1C—C9C	112.23 (8)
C5C—C6C—H6C	120.6	C11C—N1C—H13C	107.2 (11)
C6C—C5C—C4C	120.95 (9)	C9C—N1C—H13C	108.7 (11)
C6C—C5C—H5C	119.5	C11C—N1C—H14C	109.4 (11)
C4C—C5C—H5C	119.5	C9C—N1C—H14C	108.7 (10)
C2C—C3C—C4C	121.30 (10)	H13C—N1C—H14C	110.6 (15)
C2C—C3C—H3C	119.3	C5A—O4A—H1A	110.0 (12)
C4C—C3C—H3C	119.3		
			
O2B—C5B—C4B—C3B	179.66 (11)	Cl1—C1C—C2C—C3C	179.61 (8)
O3B—C5B—C4B—C3B	−1.76 (18)	C4C—C3C—C2C—C1C	0.45 (16)
C5B—C4B—C3B—C2B	−3.5 (2)	C5C—C4C—C7C—O1C	152.93 (9)
O1B—C2B—C3B—C4B	−177.70 (11)	C3C—C4C—C7C—O1C	−28.58 (13)
O4B—C2B—C3B—C4B	1.50 (18)	C5C—C4C—C7C—C10C	−91.18 (12)
O3A—C2A—C3A—C4A	0.66 (19)	C3C—C4C—C7C—C10C	87.30 (11)
O1A—C2A—C3A—C4A	−179.62 (11)	C5C—C4C—C7C—C8C	30.61 (13)
C2A—C3A—C4A—C5A	−2.4 (2)	C3C—C4C—C7C—C8C	−150.91 (9)
O2A—C5A—C4A—C3A	−176.96 (12)	C9C—C10C—C7C—O1C	−62.30 (10)
O4A—C5A—C4A—C3A	2.41 (19)	C9C—C10C—C7C—C4C	178.59 (8)
C2C—C1C—C6C—C5C	0.00 (16)	C9C—C10C—C7C—C8C	55.07 (11)
Cl1—C1C—C6C—C5C	−179.61 (8)	C11C—C8C—C7C—O1C	60.01 (11)
C1C—C6C—C5C—C4C	−0.44 (16)	C11C—C8C—C7C—C4C	−176.97 (8)
C3C—C4C—C5C—C6C	0.87 (15)	C11C—C8C—C7C—C10C	−54.84 (11)
C7C—C4C—C5C—C6C	179.36 (9)	C7C—C10C—C9C—N1C	−56.43 (11)
C5C—C4C—C3C—C2C	−0.88 (16)	C7C—C8C—C11C—N1C	56.71 (11)
C7C—C4C—C3C—C2C	−179.42 (9)	C8C—C11C—N1C—C9C	−58.48 (11)
C6C—C1C—C2C—C3C	0.00 (16)	C10C—C9C—N1C—C11C	57.97 (11)

### Hydrogen-bond geometry (Å, °)

**Table:**

*D*—H···*A*	*D*—H	H···*A*	*D*···*A*	*D*—H···*A*
O1C—H1C···O2B^i^	0.82	1.97	2.7852 (13)	171
O1A—H2A···O1B^ii^	0.90 (2)	1.68 (2)	2.5546 (13)	162 (2)
N1C—H13C···O3B^iii^	0.890 (17)	1.954 (18)	2.8328 (14)	168.6 (16)
N1C—H14C···O2A^iv^	0.887 (17)	2.087 (17)	2.9144 (15)	154.9 (15)
O4A—H1A···O3A	0.91 (2)	1.65 (2)	2.5531 (13)	173.7 (19)
O3B—H1B···O4B	1.18 (2)	1.23 (2)	2.4108 (12)	177 (2)

Symmetry codes: (i) −*x*+1/2, *y*−1/2, −*z*+1/2; (ii) *x*+1/2, −*y*+1/2, *z*+1/2; (iii) *x*, *y*−1, *z*; (iv) *x*, −*y*+1, *z*−1/2.

### Table 2 Cg···Cg interactions (Å)

Cg2 is the centroid of the C1–C6 ring.

**Table:**

*CgX*···*CgY*	Cg···Cg	*CgX*···Perp	*CgY*···Perp
Cg2···Cg2^i^	3.646 (5)	-3.460 (6)	-3.460 (6)

Symmetry code: (i) -x ,y, 1/2-z.
